# Integration of Physiological and Molecular Traits Would Help to Improve the Insights of Drought Resistance in Highbush Blueberry Cultivars

**DOI:** 10.3390/plants9111457

**Published:** 2020-10-29

**Authors:** Karen Balboa, Gabriel I. Ballesteros, Marco A. Molina-Montenegro

**Affiliations:** 1 Bachillerato en Ciencias, Facultad de Ciencias, Universidad Santo Tomás, Av. Circunvalación Poniente #1855, Talca 3460000, Chile; kbalboa@santotomas.cl; 2 Instituto de Ciencias Biológicas, Universidad de Talca, Campus Talca 3460000, Chile; 3 Centro de Estudios Avanzados en Zonas Áridas (CEAZA), Facultad de Ciencias del Mar, Universidad Católica del Norte, Coquimbo 1281, Chile; 4 Centro de Investigaciones y Estudios Avanzados del Maule (CIEAM), Universidad Católica del Maule, Talca 3460000, Chile

**Keywords:** blueberry cultivar, drought stress, water deficit tolerance index, physiological parameters, late embryogenesis abundant proteins

## Abstract

Water deficit or drought is one of the most severe factors limiting plant yield or fruit quality. Thus, water availability for irrigation is decisive for crop success, such as the case of highbush blueberry (*Vaccinium corymbosum* L.). Therefore, drought stress may compromise blueberry production due to lower fruit weight or fruit yield. Despite this, it is unclear if there is any difference in the response of blueberry cultivars to water deficit, either in terms of physiological and molecular parameters, or in terms of their sensitivity or resistance to drought. In this study, we determined the effect of drought on different physiological parameters in blueberry plants (relative water content (RWC), photochemical efficiency of photosystem II **(**Fv/Fm), Carbon Isotopic Discrimination, and proline content) in six *V. corymbosum* cultivars. We also explored molecular responses in terms of gene expression coding for late embryogenesis abundant proteins. Finally, we estimated cultivar water deficit resistance using an integrative model based on physiological results. Upon water deficit conditions, we found reductions in Fv/Fm, RWC, and isotopic discrimination of ^13^C (Δ^13^C), while proline content increased significantly for all cultivars. Additionally, we also found differences in the estimated water deficit resistance index. These results indicate differences in water deficit resistance, possibly due to variations in cultivars’ genetic composition.

## 1. Introduction

Drought stress can be defined as the absence of adequate moisture necessary for a plant to grow normally and complete its life cycle [1], and it is one of the most severe environmental factors limiting crop production [2]. Moreover, climate change models predict a global increase in temperatures, as well as reductions in rainfalls, which can cause deficient soil moisture leading to drought [3]. Drought has a negative impact on plant species of economic importance, as many physiological and biochemical processes are disturbed, causing an overall reduction in plant yield and affecting agricultural activities [4]. However, plant response to drought stress can display a wide variation among genotypes or cultivars due to gene–environment interaction [5]. Therefore, climate change increases the need to conduct screening and identification of cultivars that exhibit high resistance to drought stress, based on physiological and molecular traits as selection criterions [5,6]. This requires an understanding of which mechanisms are involved in plants’ response to water stress and drought resistance, such as restriction of leaf gas exchange, stomatal control over water loss, and establishment of a low water potential in plants [4,7].

One of the most widely used indicators of water status in plants is relative water content (RWC). This parameter reflects a balance between the absorbed water by plants (supplied to the leaf tissue) and the water lost through transpiration (transpiration rate) [8,9]. The former involves an increase in water absorption through osmotic adaptation, which enables plants to withstand drought by maintaining cell turgor and water homeostasis. This process is mediated by the production and accumulation of several osmolytes, such as proline, which provides protection to cells by decreasing osmotic potential and driving water uptake [10,11]. The reduction in water loss is achieved by stomatal closure, as plants respond to drought stress by rapidly closing their stomata due to a reduction in RWC [12]. However, stomatal closure is associated with a reduction in CO_2_ absorption available for photosynthesis [13,14]. Furthermore, water deficit can also cause an increase in premature leaf senescence and a decrease in leaf expansion [7]. Therefore, in the case of plants under water deficit conditions, photosynthesis is reduced due to a higher resistance for gas exchange and CO_2_ uptake, caused by stomatal closure and premature leaf senescence [15].

One way to estimate the efficiency of photosynthesis during drought stress is by measuring chlorophyll fluorescence parameters (such as the maximal quantum yield of photosystem II (PSII) photochemistry, F_v_/F_m_), which shows a positive correlation with leaf water potential, and has been widely used as an indicator to discriminate for drought resistant or drought sensitive plants [16]. Interestingly, the reduction in intracellular CO_2_ available for photosynthesis due to stomatal closure (in response to a decrease in leaf water potential) leads to a fixation of ^13^C by the C-fixing enzyme ribulose-1,5 bisphosphate (RuBP). This results in a plant isotopic ratio becoming less negative, and in a reduced carbon isotope discrimination (Δ^13^C) [17]. Hence, Δ^13^C is a reliable proxy for leaf-level water use efficiency (WUE) and has been used to characterize water relations of plant cultivars exposed to abiotic stress conditions [18].

In response to drought stress, plants activate different defense-responsive pathways by changing the expression levels of several regulatory and functional genes [19]. One of the most significantly induced gene families under drought stress conditions is the Late Embryogenesis Abundant (LEA) family. The LEA proteins have been linked with cellular tolerance to dehydration, as these proteins would prevent enzyme inactivation upon dehydration, participate in membrane stabilization under stress conditions, bind to water molecules, and act as oxidant scavengers of free radicals [20,21]. Interestingly, expression profile studies have shown that most of the LEA genes were highly expressed in drought resistant cultivars as opposed to drought susceptible cultivars; therefore, a resistant cultivar would have a greater ability to modulate the expression of these genes under water deficit as compared with the most susceptible cultivar [22].

However, despite the responses of plants upon water deficit conditions, a reduction in growth and a decrease in crop yield is expected [4]. This is critical in sensitive plant species with shallow root systems, such as strawberries [23], raspberries [24], and blueberries [25]. The highbush blueberry (*Vaccinium corymbosum* L.) is a deciduous shrub, whose root system is composed of fine hairless roots and mostly found in the top layers of the ground (average depth of 30 cm) [25]. Hence, it is expected that water stress would have a severe effect on this plant species [26]. Blueberry has adapted very well to a variety of soil and climatic conditions worldwide. Nevertheless, most of blueberry production is in Mediterranean climate regions, which have shown a decrease in rainfall, while climate change models predict further reduction in water availability in the coming years due to alterations in global patterns of temperature and rainfall [3,27].

Although greater resistance to water deficit is a key trait for blueberry success under such scenarios (increase in temperature and reduction in water availability), it has been seldom studied in *V. corymbosum*. In this study, we addressed the effect of water deficit on RWC, chlorophyll fluorescence, proline content, carbon isotopic discrimination, and expression levels of genes coding for LEA proteins in six different highbush blueberry cultivars. On the basis of the measured physiological activity, a water deficit resistance index model (WDTI) was constructed and used to estimate drought resistance of the examined blueberry cultivars. Hence, this study contributes to a better understanding of the effects of water deficit on blueberries and suggests which cultivars would be more resistant, opening up the possibility to select cultivars displaying resistance to drought.

## 2. Results

### 2.1. Physiological Measurements of Blueberry Plants upon Water Deficit Stress

Physiological parameters (RWC and F_v_/F_m_) were measured at 2, 4, and 6 weeks after starting the water deficit experiment. However, these parameters did not show significant differences at two or four weeks, so comparisons for all the physiological parameters used in this study were performed at six weeks (data not shown). RWC, F_v_/F_m,_ proline content, and Δ^13^C were significantly affected by irrigation, by blueberry cultivar and by the interaction effect of irrigation treatment and cultivar, except for Δ^13^C (Table 1).

**Figure 1:**
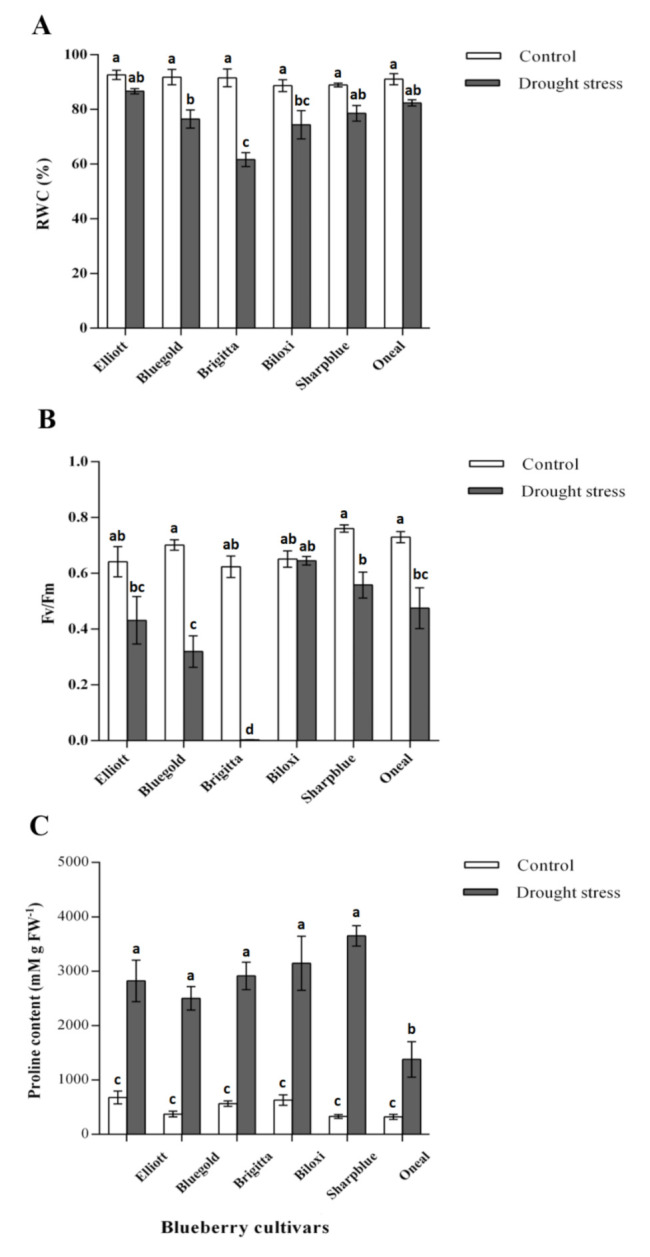
Measurements of physiological parameters after 6 weeks of drought stress. (**A**) Relative water content; (**B**) Maximum photochemical quantum yield of PSII (F_v_/F_m_); (**C**) Proline content in blueberry cultivars. White bars, control plants (watered with 300 mL) and grey bars, plants watered with 150 mL (drought stress). Data are a mean of five replicates and standard error shown as vertical bar. Significant differences were found according to two-way ANOVA and Tukey’s test (*p* value < 0.05).

In the case of RWC, water deficit conditions caused a decrease in this parameter as compared with the control conditions (Figure 1A), however, not all cultivars displayed a significant decrease. While the Brigitta cultivator had the highest RWC decrease (91.5%–61.7%), the Elliott cultivator displayed the lowest RWC decrease (92.6%–86.6%) (Figure 1A). Regarding chlorophyll fluorescence, the irrigation treatment and cultivar had a significant effect over F_v_/F_m_ (Table 1) but not all blueberry cultivars showed a significant decrease upon the water deficit conditions (Figure 1B). In the case of Bluegold, O’Neal, and Sharpblue cultivars, the measured values of F_v_/F_m_ were within the expected ranges for non-stressed plants under well-watered conditions (F_v_/F_m_ > 0.7, Figure 1B) [28]. However, in the case of the Biloxi, Elliott, and Brigitta cultivars, the F_v_/F_m_ values were slightly lower (0.65, 0.64, and 0.62, respectively). Interestingly, in plants under drought stress, Bluegold displayed the lower F_v_/F_m_ values (0.31), followed by Elliott and O’Neal (0.43 and 0.47), while Biloxi and Sharpblue displayed values of 0.64 and 0.55, respectively. Moreover, in the case of Brigitta, stressed plants displayed an extremely reduced F_v_/F_m_ value. This suggests that drought stress has a significant effect on F_v_/F_m_ in this cultivar (Figure 1B).

In all cultivars, a significant accumulation of proline (between two-fold and three-fold) was observed in water deficit conditions as compared with the control (Figure 1C). However, cultivars varied in proline accumulation levels upon water deficit; while the highest increase in proline content was measured in Sharpblue (3649.7 mM g FW^−1^ as compared with the control conditions 328.2 mM g FW^−1^) the lowest proline content was detected in O’Neal (1378.2 mM g FW^−1^) (Figure 1C).

**Figure 2:**
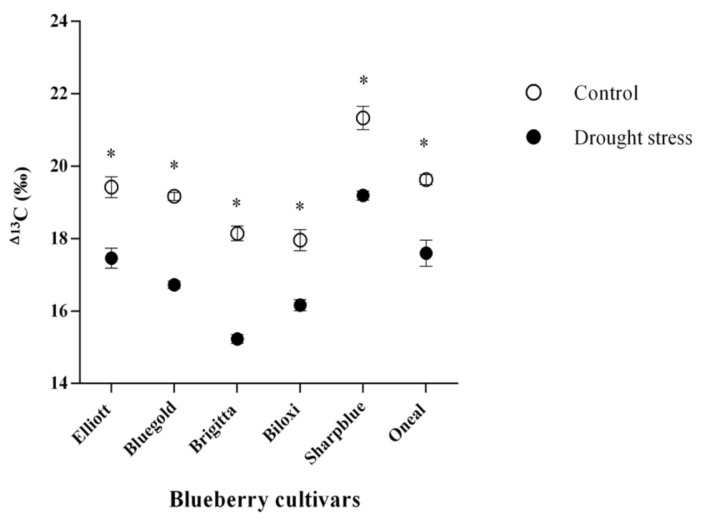
Isotopic discrimination of ^13^C (Δ^13^C) of blueberry cultivars after 6 weeks of drought stress. White circles indicate control plants (watered with 300 mL) and black circles indicate plants under drought stress (watered with 150 mL). Standard error bars are shown. * Indicate significant statistical differences between treatments for each cultivar.

Measurements of ^13^C isotopic composition (δ^13^C) in control and water deficit conditions showed that values of control plants ranged from −25.5 to −28.7 (‰), while stressed plants ranged between −22.9 and −26.6 (‰) (data not shown). Isotopic composition measurements were used as a proxy for calculation of carbon isotopic discrimination (Δ^13^C) for all cultivars and conditions, using the previously described formula. Interestingly, we found that in stress conditions, the cultivar displaying higher Δ^13^C was Sharpblue (19.1‰), while the cultivar with less discrimination was Brigitta (15.2‰) (Figure 2).

### 2.2. Integrated Estimation of Plant Blueberry Cultivar Water Deficit Resistance 

Estimation of the water deficit resistance index (WDRI) allowed us to categorization the cultivars drought resistance According to the proposed model, PPA_C_ varied from 56.76% (Sharpblue) to 51.18% (Brigitta); in the case of PPA_WD_, we observed an overall reduction, ranging from 47.05% (Biloxi) to 21.40% (Brigitta) (Table 2). Thus, and according to this estimation, Brigitta would be the worst-performing cultivar in both control and water deficit conditions. In addition, the water deficit resistance index suggests that Biloxi would display a higher water deficit resistance index (0.903) under the experimental conditions used in this study. On the other hand, Brigitta had the lowest resistance to drought (0.418). 

### 2.3. Analysis of Transcriptional Profile of Candidate Gene of the Late Embryogenesis Abundant (LEA) Family upon Water Deficit Stress

**Figure 3:**
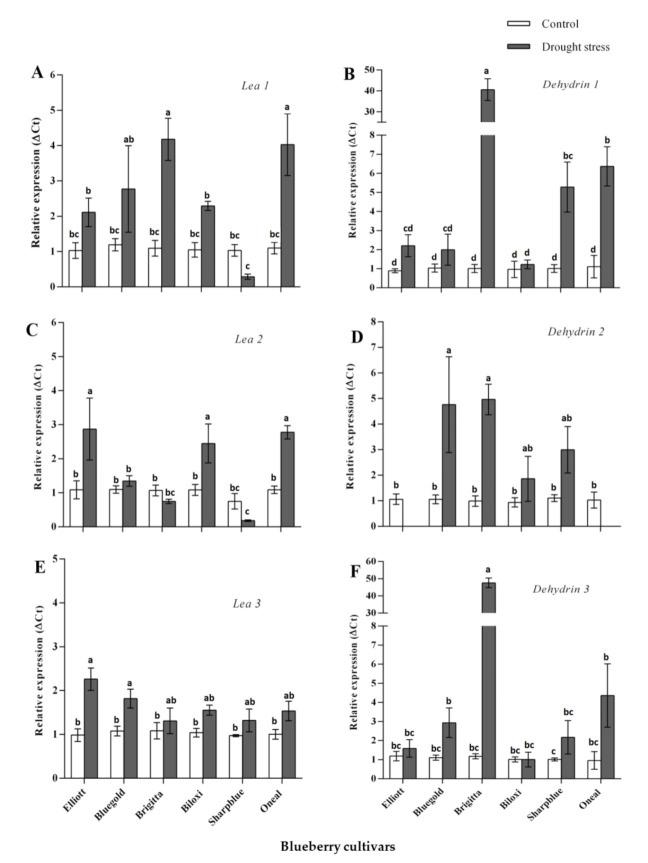
Relative expression of candidate genes from the Late Embryogenesis Abundant (LEA) family (normalized with *ACT7*) at six weeks after drought stress. (**A**): LEA 1 relative expression; (**B**): Dehydrin 1 relative expression; (**C**): LEA 2 relative expression. (**D**): Dehydrin 2 relative expression; (**E**): LEA 3 relative expression; (**F**): Dehydrin 3 relative expression. White bars, control conditions and grey bars, drought stress conditions. Standard error bars are shown. Significant differences were found according to two-way ANOVA and Tukey’s test (*p* value < 0.05).

Using bioinformatic analysis, we identified 18 candidate sequences coding for the LEA proteins in *V. corymbosum* and one sequence coding for a drought-stress marker protein (RD22). However, only six LEA-coding genes and RD22 (Supplementary Table S3) were successfully amplified by qPCR, using custom designed primers and cDNA from a pool of blueberry cultivars as a template. Thus, these genes were considered for differential expression analysis, while the other candidate genes were discarded. NormFinder analysis indicated that actin 7 (ACT7) was the most suitable reference gene for normalization of gene expression data. Hence, normalization was conducted using ACT7. We found significant diffferences in terms of relative expression levels for all candidate genes when plants were drought stressed (six weeks of drought stress) (Figure 3 and Supplementary Table S4). Our results indicate that dehydrin 1 was upregulated in all cultivars, although a higher upregulation was observed in Brigitta, Sharpblue, and O’Neal (Figure 3B). Dehydrin 2 was upregulated in four out of six cultivars (Brigitta, Sharpblue, Biloxi, and Bluegold) but no significant upregulation was observed in Elliot and O’Neal (Figure 3D). In the case of dehydrin 3, there was no significant upregulation in Biloxi as compared with the control conditions; however, upregulation was observed for the other cultivars (Figure 3F). LEA1 was not induced in Sharpblue, but it was upregulated in all the other cultivars as well (Figure 3A). In the case of LEA2, relative expression levels were higher in Elliot, Biloxi, O’Neal, and Bluegold (the latter being non-significant); however, in Brigitta and Sharpblue, no upregulation was observed (Figure 3C). LEA3 was upregulated in all cultivars (Figure 3E). Finally, RD22 was significantly upregulated in Brigitta and Biloxi; in the other cultivars, no significant differences were found. However, in Sharpblue, RD22 was slightly downregulated as compared with the control conditions (Supplementary Figure S1).

## 3. Discussion

In this study, we evaluated the response of six different highbush blueberry (*V. corymbosum*) cultivars to drought stress by simulating water deficit and measuring different physiological and molecular parameters. These results were used to estimate water deficit resistance for each blueberry cultivar through a novel “water deficit resistance index” (WDRI). This allowed us to consider the results of all measured physiological parameters, providing valuable information to compare plant physiological activity of each cultivar under water deficit conditions, resistant in terms of plants’ responses to such stress. Our experimental setup did induce a measurable response in blueberry plants for all cultivars. Furthermore, our results indicated a significant variation for several measured physiological and molecular parameters, and the WDRI model did account for such differences among blueberry cultivars (ranging from 0.418 to 0.903; Table 2). This could be explained by differences in biochemical or morpho-physiological traits, such as deeper roots systems or the ability to maintain higher plant water status [27,29], due to genetic variability (or even interspecies hybridization), as many blueberry cultivars are derived from hybrids with other blueberry species that are more resistant to drought stress (such as *V. ashei* [26]).

Leaf relative water content (RWC) is considered to be a measure of plant water status, as it impacts on metabolic activity in tissues and has been used as an indicator of the level of tissue dehydration [30]. In the present study, water deficit treatment caused a significant reduction in RWC for three cultivars. On the one hand, a possible explanation could be that RWC is related to water uptake by the roots, as well as water loss by transpiration [30]. However, blueberry has a shallow root system, which would not be able to compensate for the water lost by transpiration [27,30]. Furthermore, it is plausible that the significant differences in RWC found among cultivars could be due to variations in root mass and root/shoot dry weight ratio among studied cultivars. Therefore, blueberry cultivars with the deepest root systems or higher root mass would require less frequent irrigation than other cultivars for optimum growth and production [29]. On the other hand, genotypic differences among the blueberry cultivars could also be responsible for the observed RWC significant differences, as studies conducted in other plant species (such as wheat) have shown significant differences in RWC among wheat genotypes [8]. In the case of sugarcane, drought-resistant genotypes would maintain a better leaf water relation in terms of RWC as compared with drought-susceptible varieties under water deficit conditions [31]. Taken together, and according to the RWC measurements, Brigitta would be a drought susceptible cultivar, while Elliot would be drought resistant, as it is able to maintain a relatively high RWC during water deficit.

Photosynthesis is essential in the maintenance of plant growth and development, but is one of the key processes affected by drought stress in higher plants, due to a reduction in CO_2_ assimilation caused by stomatal closure on plant leaves [32]. In this context, carbon isotopic discrimination (Δ^13^C) has been identified as a potentially useful proxy for estimating the trade-off between photosynthesis and transpiration, reflected by water use efficiency (WUE). Therefore, plants with higher WUE should have reduced Δ^13^C values and would display reduced drought resistance tolerance [33]. Additionally, a reduction in water loss through transpiration (due to stomatal closure) has been related to an impairment of the photosynthetic apparatus. One of the most important indicators of photosynthetic efficiency is the maximum quantum yield of photosystem II (F_v_/F_m_) [16,32].

In our study, water deficit conditions caused a significant decrease in F_v_/F_m_ values for all blueberry cultivars, except for Biloxi. In this cultivar, no significant differences were found as compared with the well-watered conditions, and it maintained a F_v_/F_m_ value similar to the control conditions (~0.64), indicating a minor impairment of the photosynthetic apparatus [32]. Indeed, in rapeseed plants (*B. napus*) under water deficit stress, the cultivars with higher F_v_/F_m_ ratios demonstrated a clearly higher drought resistance, suggesting that maximum quantum yield of photosystem II could be an effective selection criterion in screening for crop plants with drought resistance [16]. Regarding Δ^13^C, we observed an overall reduction under water deficit conditions for all blueberry cultivars, which indicated that all cultivars were fixating ^13^C by ribulose 1,5-bisphosphate (RuBP). However, a high variability was observed among blueberry cultivars in terms of Δ^13^C as comparing with the well-watered conditions (control) or water-deficit conditions. According to our results, on the one hand, Brigitta showed the lowest Δ^13^C, thus, it was more efficient in water usage and would be a drought sensitive cultivar. On the other hand, Biloxi showed the least reduction in terms of Δ^13^C, suggesting that this cultivar would be less affected by the water deficit conditions used in this study.

Taken together, these results point towards an overall reduction in photosynthetic efficiency and a decrease in Δ^13^C in blueberry, due to water deficit. However, not all cultivars displayed similar responses to drought stress in terms of F_v_/F_m_ or Δ^13^C discrimination; according to these results, Brigitta would be the most drought sensitive cultivar, while Biloxi would be the most drought resistant. Hence, it could be possible that genetic differences among cultivars would explain the observed differences in water deficit [34]. Therefore, both F_v_/F_m_ and Δ^13^C appear to be accurate parameters for screening blueberry cultivars in terms of drought sensitivity/resistance [35].

Among the molecular mechanisms involved in response to drought stress are the accumulation of the LEA proteins and compatible osmolytes, such as prolines. The LEA proteins have been correlated with the ability to survive the removal of a significant proportion of cellular water without irreversible damage (desiccation tolerance) [36]. For example, the LEA group II proteins (dehydrins) play several roles such as chaperone activity, membrane protection, cryoprotection, and resistance to abiotic stress [37]. In the case of the LEA III and LEA IV proteins, an increased accumulation of these proteins/transcripts has been correlated to events of abiotic stress (cold, saline, and drought), although their precise functions remain redox homeostasis [38,39]. Furthermore, in many plants, a strong correlation unclear [40]. Proline accumulation is associated with an improvement in drought resistance, as it participates in stress-protective functions, both as a compatible osmolyte and as a metabolic signal that regulates the stabilization of macromolecules, proteins, antioxidant enzymes, and the balance of intracellular has been established between proline accumulation and drought stress resistance [41].

In this study, we found changes in the expression levels for three genes coding for dehydrins, and two genes coding for the LEA III and LEA IV proteins, although we did not find a clear pattern of gene up- or downregulation in the tested cultivars. Despite that Brigitta had the higher transcript levels of dehydrins and *LEA1* as compared with the other cultivars under water deficit, suggesting that these proteins may be playing an important role in response to drought stress [42], we did not find a relationship with plant physiological activity. Therefore, it could be possible that LEA proteins may alleviate the damage caused by water deficit in this cultivar [42]. Overall, these results suggest that different cultivars would induce different LEA proteins in response to drought stress, as the occurrence of more than one type of LEA protein in a single organism suggests multiple subcellular locations and the ability to perform divergent functions [43]. A possible explanation for this phenomenon would be the differences in terms of genetic variability/composition among blueberry cultivars, which could explain the observed transcriptional patterns of the studied *LEA* coding genes. Alternatively, these genes could be expressed earlier in response to drought stress conditions [22]. Regarding proline accumulation, we found an increase in proline content in plants under water deficit conditions, suggesting that this osmolyte would act as an osmoprotectant in blueberry during this stress [44]. Despite this, the level of accumulated proline is rather the same as in resistant cultivars (Figure 1C and Table 2), which suggests that it would not have a significant role in drought resistance in the examined blueberry cultivars. Therefore, it could be possible that these cultivars use an alternate strategy to cope with drought stress (such as the accumulation of other compatible solutes), rather than accumulation of proline. Hence, LEA protein expression levels could give useful insights regarding putative molecular mechanisms involved in response to drought stress in blueberry cultivars; however, further studies should be conducted in order to use these traits in breeding programs aimed at selecting resistant tolerant blueberry cultivars to drought stress.

## 4. Conclusions

In this study, we found that Biloxi displayed a small reduction in the measured Plant Physiological Activity under water deficit conditions compared to control (47.05 vs. 52.14), although it did not show the highest measured physiological activity as compared with the other cultivars in control conditions. Furthermore, and using the Water Deficit Resistance Index, we found that Biloxi is the cultivar displaying the higher resistance to drought stress. This suggests that Biloxi could possibly be following a “jack-of-all-trades” strategy, as, on the one hand, it would be most successful under drought stress conditions used in this study (e.g., by maintaining similar fitness) [45]. However, this would also come at the cost of reduced physiological performance in well-watered conditions. On the other hand, and according to PPA and WDRI, Brigitta would be the worst performing cultivar, even in well-watered conditions. Nonetheless, we propose to evaluate more cultivars and drought stress indicators using drought stress resistance/sensitivity assays to make a robust water deficit resistance index, as it is extremely difficult to predict a priori drought stress resistance or susceptibility of a particular cultivar based only on cultivar genotype composition [25]. We also propose to include other important characteristics, such as fruit yield and nutritional quality, in breeding programs aiming at developing drought-tolerant cultivars. Finally, because further reductions in water availability are to be expected due to climate change, studies should be conducted in order to determine which blueberry cultivar/genotype would have a better performance in a specific geographic/climatic location.

## 5. Materials and Methods

### 5.1. Plants, Growth Conditions, and Drought Treatment

The following six blueberry cultivars were evaluated: Elliot, Bluegold, Brigitta, Biloxi, Sharpblue, and O’Neal (Supplementary Table S1). The plant material was propagated in culture medium containing Woody Plant Medium [46], Murashige & Skoog vitamins (MS) [47], and supplemented with sucrose and 2-iP (6-(y,y-dimethylallylamino)purine) as a growth regulator. The pH was adjusted to 5.2 with NaOH or HCl, before sterilization to 121 °C, for 20 min. Blueberry seedlings (from tissue culture) were transferred to a transplant tray filled with growing medium (peat/perlite substrate, 4:1) for acclimatization in a growth chamber (23 °C, D16/N8 photoperiod). Then, ten plants for each cultivar were transferred to plastic pots of 800 mL filled with the same growth medium and watered periodically. Half-strength Hoagland solution was applied biweekly for one year before conducting the experiments. All plants were maintained in the same greenhouse where experiments were conducted.

Water volume for irrigation used in both control and stress conditions was estimated through the relative moisture content of substrate, by measuring substrate electrical conductivity [48] in 5 pots filled with the same medium and with presence of a single blueberry plant randomly chosen. The following five different irrigation volumes were evaluated: 100, 150, 200, 250, and 300 mL, which were applied at 48 h. Probes (ECHO-R2, Onset, Bourne, MA, USA) were used to register electrical conductivity for a week, and enabled soil moisture content estimation. This technique is based on the negative correlation between the electrical resistance of the soil and its water content, i.e., as the water soil content decreases, its electrical resistance increases [48]. Considering these results, an irrigation volume of 300 mL was used for the control conditions and 150 mL for the deficit water conditions, every other day. With these volumes, it was possible to obtain significant differences between treatments. The experiment was carried out in a greenhouse at Talca University (Talca, Chile), during the 2017 summer season (December 2016–January 2017). All plants were irrigated with 300 mL for two weeks every other day and were in the same phenological state before starting the water deficit experiment. For each cultivar, plants were randomly divided in two groups and were either assigned to the control or the water deficit conditions (*n* = 5 per treatment/cultivar). Tissue sample collection and measurements were performed at the beginning of the experiment, and at 2, 4, and 6 weeks after water deficit experiment.

### 5.2. Physiological Measurements of Blueberry Cultivars upon Water Deficit Stress

#### 5.2.1. Relative Water Content

For relative water content (RWC) measurements, one fully expanded leaf sample was collected from each individual. RWC was calculated using the following formula [31]:

(fresh weight − dry weight)/(turgid weight − dry weight) × 100 (1)

Leaf fresh weight was measured immediately after sampling, while turgid weight was measured after leaf hydration in deionized water for 24 h in room temperature. Then, samples were oven-dried at 65 °C, for 48 h, to determine dry weight [31].

#### 5.2.2. Chlorophyll Fluorescence

Photochemical efficiency of PSII during water deficit stress was estimated by using leaf chlorophyll fluorescence measurements, which were carried out using a portable pulse-amplitude-modulation fluorometer (Pocket PEA, Hansatech Instruments, UK). The chlorophyll fluorescence ratio F_v_/F_m_ (F_v_ = F_m_ − F_0_) was used to detect changes induced by drought conditions in the maximum quantum yield of photosystem II (PSII). Measurements were conducted at noon on fully expanded leaves and were carried, after 20 min of leaf dark adaptation, by means of clip leaf for both control and treatment plants.

#### 5.2.3. Leaf Proline Content

To evaluate proline accumulation during drought stress conditions in blueberry cultivars, proline content was analyzed using the modified procedure of Bates et al. [49] from fully expanded leaves collected for both control and treatment plants. Approximately 100 mg of leaf tissue were ground in liquid nitrogen, homogenized with 2 mL of 3% sulfosalicylic acid in 15 mL tubes, and centrifuged at 7000 rpm for 15 min, at 4 °C. One ml of extract supernatant was reacted with 1 mL of ninhydrin acid and 1 mL of glacial acetic acid, boiled in a hot water bath at 100 °C for 45 min, and incubated for 30 min at 0 °C. Then, 1.5 mL of toluene was added to each tube and centrifuged for 10 min at 7000 rpm. The toluene fraction was extracted, and absorbance was measured at 520 nm in a spectrophotometer (Jenway 6300, Cole-Parmer, Staffordshire, UK). The amount of free proline was determined using a standard curve and expressed as mM g^−1^ tissue fresh weight.

#### 5.2.4. Carbon Isotope Discrimination (Δ^13^C)

Carbon isotope composition (δ^13^C*)* (a proxy for estimation of water use efficiency) was assessed on fully expanded leaves samples which were homogenized and oven-dried, at 60 °C, for 72 h. Dried samples were sent to the Stable Isotopes Laboratory (Montana University) for isotope analysis. Briefly, samples were weighted and combusted in tin capsules (IVA Analysentechnik, Meerbusch, Germany) in an elemental analyzer coupled to an isotope ratio mass spectrometer. The isotopic composition of ^13^C was calculated using the following formula:

(2) $$\delta^{13}C\left(‰\right)=\left(\frac{R_{sample}}{R_{standard}}-1\;\right)$$

where standard is Pee Dee Belemnite calcium carbonate (PDB) [50]. Isotopic discrimination was calculated using the following formula:

(3) $$\Delta^{13}C\left(‰\right)=\left(\frac{\delta^{13}C_{a}-\delta^{13}C_{p}}{1+\delta^{13}C_{p}}\;\right)$$

where δ^13^C*_a_* is the carbon isotope signature of the air (−8‰ [51]) and δ^13^C*_p_* is the isotopic signature of the plant; carbon isotope data is expressed in Δ^13^C (per mil ‰).

### 5.3. Integrated Estimation of Plant Blueberry Cultivar Physiological Activity

In order to provide an overall estimation of the physiological activity for each cultivar, we devised an ad hoc equation representing the “physiological plant activity” (PPA) (3), which was used as input to a “water deficit resistance index” (WDRI) (4) [52]. PPA equation used as input the absolute values of RWC, F_v_/F_m_, and δ^13^C, which were identically weighted. As this equation used a scale of 0 to 1 for each parameter, RWC and δ^13^C values were converted to decimal values, by dividing RWC by 100 and δ^13^C by 1000. Thus, the following model was used for plant physiological activity and water deficit resistance index:

PPA = [(0.333 × RWC) + (0.333 × Fv/Fm) + (0.333 × δ13C)] × 100(4)

RI = PPAWD/PPAC(5)

Where PPAC = Plant Physiological Activity in control condition

PPAWD = Plant Physiological Activity in Water Deficit conditions

Therefore, WDRI values ~0 indicate that the blueberry cultivar had a lower resistance to drought, while cultivars with WDRI values ~1 indicate a higher resistance to drought stress conditions.

### 5.4. Analysis of Transcriptional Profiles of the LEA Family Candidate Genes upon Water Deficit Stress

#### 5.4.1. In Silico Analysis and Primer Design

To evaluate expression levels of the LEA candidate genes, we searched for *V. corymbosum* nucleotide sequences coding for these proteins using several public databases, including two annotated blueberry transcriptome sequencing projects [53,54] and GenBank, NCBI (both in Nucleotide database and dbEST; [55]). In the case of ESTs, we performed an alignment using BLASTx ver 2.7.0 against the NCBI NR database to screen for genes coding for LEA proteins; additionally, the annotation of candidate genes from other databases was verified by using BLASTx and using conserved domain database (Supplementary Table S2). We also selected a widely used drought-stress marker gene coding for a dehydration-responsive protein (*RD22*) which was induced under water deficit conditions [56]. Finally, for each selected gene, specific primer pairs (listed in Supplementary Table S3) were designed using Beacon Designer 8.12 (Premier Biosoft, San Francisco, CA, USA).

#### 5.4.2. RNA Isolation from Blueberry Leaves, cDNA Synthesis, and Gene Expression Analysis

For RNA isolation, 1–2 g of fully expanded leaf samples were collected separately from five different plants (five biological replicates) for each blueberry cultivar and conditions (control or water deficit). RNA was obtained by using the modified perchlorate method (5M sodium perchlorate, 300 mM Tris-Hcl pH 8, 1% v/v SDS, 2% v/v PEG 20,000, 8.5% p/v PVPP, and 3% v/v 2-mercaptoethanol) [57]. RNA sample integrity was assessed using a 1.1% denaturing formaldehyde agarose gel electrophoresis, while concentration and purity was estimated by spectrophotometry at 260 nm and OD260/280 ratio > 1.8 (Epoch Microplate Spectrophotometer, Biotek, VT, USA). DNA traces were removed from total RNA by DNAse treatment using Ambion^®^ TURBO DNase (Thermo Fisher Scientific, Waltham, MA, USA). Single-stranded cDNAs synthesis was carried out from 2 ug of total RNA for each sample using oligo(dT), according to the manufacturer’s instructions (Maxima H Minus First Strand cDNA Synthesis Kit, Thermo Fisher Scientific, Waltham, MA, USA). These cDNAs were, in turn, used to determine the relative transcript abundance of the LEA coding genes by real-time PCR (qPCR) (primers described in Supplementary Table S3). Each qPCR reaction contained 2 μL of diluted cDNA (50 ng), 10 μL Maxima SYBR Green PCR Master Mix (Thermo Fisher Scientific, Waltham, Massachusetts, USA), 6.4 μL of nuclease free water, and 0.8 μL of each specific primer (1.6 μL for both forward and reverse primers, 10 mM concentration). Negative controls (nuclease-free water) were included for detecting any cross-contamination; positive controls for qPCR reactions were also included (*V. corymbosum* genomic DNA). All PCR reactions were carried out in triplicate using the Mx3000P qPCR system (Agilent, Santa Clara, CA, USA) under the following cycling conditions: 95 °C for 10 min, 40 cycles of 95 °C for 30 s, 60 °C for 60 s, and 72 °C for 20 s [58]. A dissociation curve was included immediately after each qPCR using a ramp of 55–95 °C to confirm the absence of nonspecific amplifications. Each PCR reaction was performed in triplicate (three technical replicates) and the mean of five biological replicates was calculated. Three normalizer genes were used in this study: *polyubiquitin 3* (*UBQ3b*), *actin 7* (*ACT7*), and *glyceraldehyde-3-phosphate dehydrogenase* (*GAPDH*) described for *V. corymbosum* [58,59] (primers in Supplementary Table S3). Because there was no information regarding expression stability available for blueberry, we used NormFinder [60] to determine expression stability of these reference genes and to identify a suitable normalizer gene for blueberry plants under drought stress. For each target gene, relative expression levels were calculated using the comparative 2^−ΔΔCT^ method between the control and water deficit conditions plants [61].

### 5.5. Statistical Analysis

The experiment was carried out with six blueberry cultivars, using five replicates per treatment (control and water deficit conditions) in a completely random block design. Statistical differences between conditions were determined using two-way ANOVA, followed by a Tukey’s test (*p* ≤ 0.05). The independent variables were blueberry cultivars and conditions (control or water deficit) while the dependent variables were the parameters measured (RWC, Fv/Fm, proline content, and relative expression of candidate genes). The statistical software package STATISTICA 8.0 (StatSoft, Tulsa, OK, USA) was used for data analysis.

## Figures and Tables

**Table 1:** Two-way ANOVA of the interactions between irrigations (300 mL and 150 mL) and relative water content (RWC), photochemical efficiency of photosystem II (F_v_/F_m_), proline content, and ^13^C isotopic composition in different blueberry cultivars. Significant *p-*values (*p* < 0.05) are shown in bold.

Source of Variation	d.f	MS	F	*p*
**Relative water content (RWC)**				
Intercept	1	403,822.9	13,284.36	*p* < 0.001
Cultivar	5	184.7	6.08	*p* < 0.001
Irrigation	1	2855.2	93.93	*p* < 0.001
Cultivar*Irrigation	5	173.3	5.70	*p* < 0.001
Error	46	30.4		
**Photochemical efficiency of PSII (F_v_/F_m_)**				
Intercept	1	20.89461	1607.872	*p* < 0.001
Cultivar	5	0.17368	13.365	*p* < 0.001
Irrigation	1	1.20068	92.394	*p* < 0.001
Cultivar*Irrigation	5	0.11042	8.497	*p* < 0.001
Error	60	0.01300		
**Proline content**				
Intercept	1	155,304,002	946.2628	*p* < 0.001
Cultivar	5	1,726,505	10.5195	*p* < 0.001
Irrigation	1	76,087,947	463.6017	*p* < 0.001
Cultivar*Irrigation	5	1,340,468	8.1674	*p* < 0.001
Error	48	164,124		
**Carbon Isotopic Discrimination of ^13^C**				
Intercept	1	38,045.46	181,554.1	*p* < 0.001
Cultivar	5	14.85	70.9	*p* < 0.001
Irrigation	1	64.67	308.6	*p* < 0.001
Cultivar*Irrigation	5	0.38	1.8	0.1302
Error	46	0.21		

**Table 2:** Physiological plant activity and water deficit resistance index (%) calculated using the proposed model (3,4) measured at week 6 after drought stress treatment (RWC, F_v_/F_m_, and δ^13^C). * indicates best performing cultivar under drought.

Cultivar	PPA Control (%)	PPA Water Deficit (%)	WDRI (PPA_WD_/PPA_C_)
Sharpblue	56.76	45.63	0.804
O’Neal	55.77	44.10	0.791
Bluegold	54.56	36.91	0.677
Elliott	53.11	44.05	0.830
Biloxi *	52.14	47.05	0.903
Brigitta	51.18	21.40	0.418

## Supplementary Materials

The following are available online at https://www.mdpi.com/2223-7747/9/11/1457/s1, Figure S1: Relative expression analysis of *RD22* (normalized with *ACT7*) of six *V. corymbosum* cultivars at six weeks after drought stress. White bars, control conditions and grey bars, drought stress conditions. Standard error bars are shown. * indicate significant statistical differences between treatments, Table S1: Blueberry cultivars used in this study. The pedigree of each cultivar is listed from https://www.ars-grin.gov/cor/catalogs/vacblue.html, and genetic composition is also indicated (Lobos et al., 2015; Rowland et al., 2003). VC, *Vaccinium corymbosum*; VA, *V. angustifolium*; VD, *V. darrowii*; Vas, *V. ashe**i*, Table S2: Annotation and conserved domain classification of candidate genes from LEA superfamily used in this study for qPCR expression analysis. **§**, sequences available in GenBank; †, sequences derived from Darwish et al. (2013) (http://bioinformatics.towson.edu/BBGD454/Default.aspx); δ, sequences derived from Gupta et al. (2015) (https://bitbucket.org/lorainelab/blueberrygenome), Table S3: Nucleotide sequences of primers employed for qPCR in this study (LEA and normalizer genes). §, sequences available in GenBank; †, sequences derived from Darwish et al. (2013) (http://bioinformatics.towson.edu/BBGD454/Default.aspx); δ, sequences derived from Gupta et al. (2015) (https://bitbucket.org/lorainelab/blueberrygenome). * Indicates primer sequences reported in Walworth et al., 2012 (*), Zifkin et al., 2012 (**) and Vashisth et al., 2011 (***). All primer pairs amplify the 3′ UTR region of each selected gene, have a melting temperature of 59 °C ± 1, and a predicted amplicon length of 60–200 bp, Table S4: Two-way ANOVA of the interaction between irrigations (300 mL and 150 mL) at six weeks of drought stress for candidate genes coding for the LEA family proteins in different blueberry cultivars. Significant *p-*values (*p* < 0.05) are shown in bold.
