# Supplementary material for: Integration of Physiological and Molecular Traits Would Help to Improve the Insights of Drought Resistance in Highbush Blueberry Cultivars
Source: Plants (Basel). 2020 Oct 29;9(11):1457. doi: 10.3390/plants9111457 (PMC7693893; doi:10.3390/plants9111457)
Supplement: Supplementary file 1 [file plants-09-01457-s001.pdf]

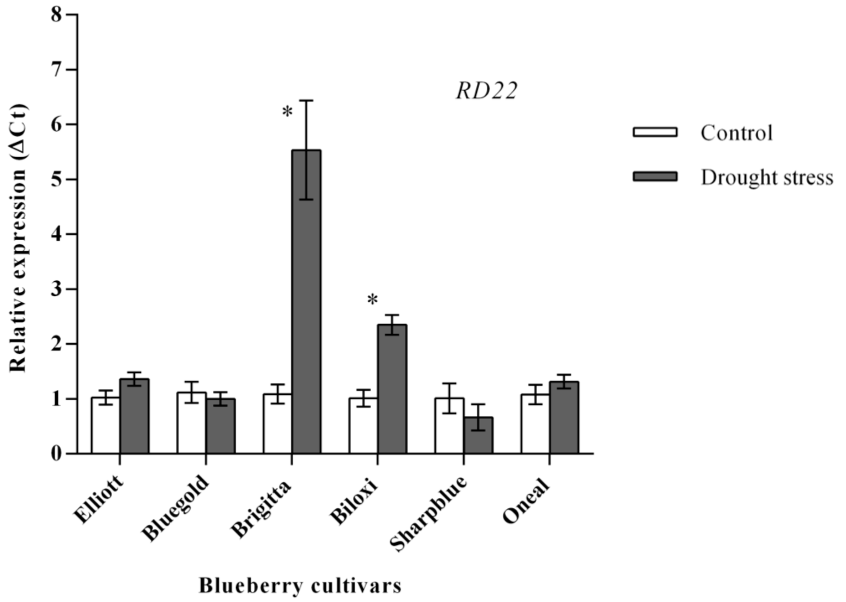

Supplementary Figure 1. Relative expression analysis of *RD22* (normalized with *ACT7*) of six *V. corymbosum* cultivars at six weeks after drought stress. White bars: control condition. Grey bars: drought stress condition. Standard error bars are shown. * indicate significant statistical differences between treatments.

Supplementary Table 1. Blueberry cultivars used in this study. The pedigree of each cultivar is listed from https://www.ars-grin.gov/cor/catalogs/vacblue.html, and genetic composition is also indicated (Lobos et al., 2015; Rowland et al., 2003). VC: *Vaccinium corymbosum*; VA: *V. angustifolium*; VD: *V. darrowii*; Vas: *V. ashei.*

Blueberry Cultivar	Pedigree	Genetic species composition (%)
Elliott	Burlington x US 1 [Dixi x (Jersey x Pioneer)]	100 VC
Bluegold	Bluehaven x Me-US 5 (Ashworth x Bluecrop)	89,5 VC<br>14,1 VA
Brigitta	open pollinated Lateblue (at Michigan State University) selected in Australia	100 VC
Biloxi	Sharpblue x [US210(US67 x US132) x Florida 4-76(Bluecrop x13-236)]	41,8 VC<br>1,8 VA<br>32,5 VD<br>11,3 Vas
Sharpblue	*V. corymbosum* x *V. asheii* & *V. darrowi* (Fla 61-5 x Fla 62-4) tetraploid	43,7 VC<br>28,8 VA<br>15 VD<br>12,5 others
O'Neal	Wolcott x Fla. 4-15 mainly *corymbosum*, some *angustifolium*, *ashei*, *darrowi*	84 VC<br>10 VA<br>3 VD<br>3 Vas

Supplementary Table 2. Annotation and conserved domain classification of candidate genes from LEA superfamily used in this study for qPCR expression analysis. §: sequences available in GenBank. ✝: sequences derived from Darwish et al. (2013) (http://bioinformatics.towson.edu/BBGD454/Default.aspx). ẟ: sequences derived from Gupta et al. (2015) (https://bitbucket.org/lorainelab/blueberrygenome).

Candidate gene	Annotation	Conserved domain classification	E-value	Reference ID
Dehydrin 1	Dehydrin 1 (bbdhn1)	Dehydrin superfamily	7.5e-19	AF030180.1§
Dehydrin 2	COR11 (bbdhn7)	Dehydrin superfamily	2.3e-17	AY660960.1§
Dehydrin 3	14-kDa dehydrin (bbdhn6)	Dehydrin superfamily	2.3e-03	AY660959.1§
LEA1	Late embryogenesis abundant protein Dc3	Late embryogenesis abundant protein LEA4	1.0e-019	CUFF.38534.1ᵟ
LEA2	Late embryogenesis abundant protein Lea5	Late embryogenesis abundant protein LEA3	8.3e-20	contig07652†
LEA3	Late embryogenesis abundant protein	Laminin Domain II	3.4e-03	contig13365†
RD22	Dehydration-responsive protein RD22	BURP (RD22) containing domain protein	9.72e-93	gene.g32217.t1.1ᵟ

Supplementary Table 3. Nucleotide sequences of primers employed for qPCR in this study (LEA and normalizer genes). §: sequences available in GenBank. †: sequences derived from Darwish et al. (2013) (http://bioinformatics.towson.edu/BBGD454/Default.aspx). ᵟ: sequences derived from Gupta et al. (2015) (https://bitbucket.org/lorainelab/blueberrygenome). * indicates primer sequences reported in Walworth et al., 2012 (*), Zifkin et al., 2012 (**) and Vashisth et al., 2011 (***). All primer pairs amplify the 3′- UTR region of each selected gene, have a melting temperature of 59°C±1, and a predicted amplicon length of 60-200 bp.

Gene	Reference ID	Primer sequence
Dehydrin 1	AF030180.1§	FW-5'- GGTGGTGGTGCTGATAAG-3'<br>RV-5'-GACGTGATGATGAATCAATCTAAT-3'
Dehidrin 2	AY660960.1§	FW-5'- ATC TCT AGG TGT GGA CAG-3'<br>RV-5'- AGC ACT CTT CTC ATT TCT TT-3'
Dehydrin 3 (*)	AY660959.1§	FW-5’-CGCGGCGATTAGATCGAA-3’<br>RV-5’-AGCTAGCGTAGAGGCGGAAA-3’
LEA 1	CUFF.38534.1 ᵟ	F-5'-CACATTATTGTTGTCGTACTAT-3'<br>R-5'- ACAAGATAGAAGGGAATCAC-3'
LEA 2	contig07652†	F-5'- GATAAACACCAAAACTGAAGA-3'<br>R-5'- GGTCAAATCCAAACAAATATAAC-3'
LEA 3	contig13365†	F-5'-GTTTGTTTGTAACTCCATCA-3'<br>R-5'-TTAAATATCAGAAACCAAGGTAC-3'
RD22	gene.g32217.t1.1ᵟ	FW-5’- CCACAAGACCACCACCACAA-3’<br>RV-5’- ACACAGCCACTGCCTTAGC-3’
GAPDH (**)	-	FW- 5’-GGTTATCAATGATAGGTTTGGCA-3’<br>RV-5’- CAGTCCTTGCTTGATGGACC-3’
UBQ3b (***)	-	FW-5’-CCTCCACTTGGTGCTCCGT-3’<br>RV-5’-AGATGAGCCTCTGCTGATCCG-3’
ACT7 (***)	-	FW-5’-TGGTCCATCCATTGTCCACAGGAA-3’<br>RV-5’-TATGCCCTTCCAGTTGCAATCACC-3’

Supplementary Table 4. Two-way ANOVA of the interaction between irrigation (300 ml and 150 ml) at six week of drought stress for candidate genes coding for LEA family proteins in different blueberry cultivars. Significant *p*-values ($p < 0.05$) are shown in bold.

Source of variation	d.f	MS	F	*P*
		Dehydrin 1		
**Cultivar**	5	815,846	43,1040	**0,000000**
**Irrigation**	1	1415,852	74,8045	**0,000000**
**Cultivar*Irrigation**	5	814,214	43,0178	**0,000000**
**Error**	68	18,927		
		Dehydrin 2		
**Cultivar**	5	18,2801	2,76101	**0,023759**
**Irrigation**	1	34,5245	5,21453	**0,025086**

**Cultivar*Irrigation**	5	18,8455	2,84640	**0,020483**
**Error**	79	6,6208		
Dehydrin 3				
**Cultivar**	5	1166,894	137,9216	**0,00**
**Irrigation**	1	1550,346	183,2438	**0,00**
**Cultivar*Irrigation**	5	1155,570	136,5831	**0,00**
**Error**	69	8,461		
Lea 1				
**Cultivar**	5	7,0443	2,8731	**0,019240**
**Irrigation**	1	52,2802	21,3234	**0,000014**
**Cultivar*Irrigation**	5	6,5206	2,6596	**0,027950**
**Error**	83	2,4518		
Lea 2				
**Cultivar**	5	3,8766	5,2142	**0,000438**
**Irrigation**	1	7,4925	10,0779	**0,002294**
**Cultivar*Irrigation**	5	2,7617	3,7147	**0,005099**
**Error**	65	0,7435		
Lea 3				
**Cultivar**	5	0,4448	1,6833	0,148833
**Irrigation**	1	7,6417	28,9190	**0,000001**
**Cultivar*Irrigation**	5	0,5100	1,9300	0,099107
**Error**	76	0,2642		
RD22				
**Cultivar**	5	8,3003	11,1114	**0,000000**
**Irrigation**	1	18,7656	25,1208	**0,000005**
**Cultivar*Irrigation**	5	7,7061	10,3159	**0,000000**
